# A Pilot Study Comparing the Efficacy of Lactate Dehydrogenase Levels Versus Circulating Cell-Free microRNAs in Monitoring Responses to Checkpoint Inhibitor Immunotherapy in Metastatic Melanoma Patients

**DOI:** 10.3390/cancers12113361

**Published:** 2020-11-13

**Authors:** Matias A. Bustos, Rebecca Gross, Negin Rahimzadeh, Hunter Cole, Linh T. Tran, Kevin D. Tran, Ling Takeshima, Stacey L. Stern, Steven O’Day, Dave S. B. Hoon

**Affiliations:** 1 Department of Translational Molecular Medicine, John Wayne Cancer Institute (JWCI), Providence Saint John’s Health Center (SJHC), Santa Monica, CA 90404, USA; Rebecca.Gentry@providence.org (R.G.); Negin.Rahimzadeh@providence.org (N.R.); TakeshimaL@jwci.org (L.T.); HoonD@jwci.org (D.S.B.H.); 2 Department of Immuno-Oncology and Clinical Research, JWCI, Providence SJHC, Santa Monica, CA 90404, USA; ColeH@jwci.org (H.C.); O'DayS@jwci.org (S.O.); 3 Department of Genomic Sequencing Center, JWCI, Providence SJHC, Santa Monica, CA 90404, USA; Linh.Tran3@providence.org (L.T.T.); Kevin.Tran@providence.org (K.D.T.); 4 Department of Biostatistics, JWCI, Providence SJHC, Santa Monica, CA 90404, USA; SternS@jwci.org

**Keywords:** serum LDH, blood biomarker, miRNA, circulating microRNA, plasma, immunotherapy, immune checkpoint inhibitors, metastatic melanoma

## Abstract

**Simple Summary:**

Improvement in melanoma patients with metastatic disease is needed to better assess immunotherapies. Lactate dehydrogenase (LDH) is currently an accepted biomarker for stage IV, but it has limited utility for stage III melanoma patients. Thus, finding biomarkers for metastatic melanoma is important not only to identify progressive melanoma tumors, but also to monitor patients under checkpoint inhibitor immunotherapy (CII). The aim of this pilot study was to demonstrate the utility of circulating cell-free microRNAs (cfmiRs) as potential blood biomarkers for stage III and IV melanoma patients compared to LDH. To accomplish this aim, we profiled for cfmiR the plasma of metastatic melanoma patients before and during CII treatment, and compared them to normal healthy donors’ samples. The cfmiR profiling was performed using an NGS-based miRNA assay, which requires no extraction and a small volume input. We found specific cfmiR signatures in stage III and IV metastatic melanoma patients. As a proof of concept, our results showed that certain cfmiRs are associated with CII outcomes.

**Abstract:**

Serum lactate dehydrogenase (LDH) is a standard prognostic biomarker for stage IV melanoma patients. Often, LDH levels do not provide real-time information about the metastatic melanoma patients’ disease status and treatment response. Therefore, there is a need to find reliable blood biomarkers for improved monitoring of metastatic melanoma patients who are undergoing checkpoint inhibitor immunotherapy (CII). The objective in this prospective pilot study was to discover circulating cell-free microRNA (cfmiR) signatures in the plasma that could assess melanoma patients’ responses during CII. The cfmiRs were evaluated by the next-generation sequencing (NGS) HTG EdgeSeq microRNA (miR) Whole Transcriptome Assay (WTA; 2083 miRs) in 158 plasma samples obtained before and during the course of CII from 47 AJCC stage III/IV melanoma patients’ and 73 normal donors’ plasma samples. Initially, cfmiR profiles for pre- and post-treatment plasma samples of stage IV non-responder melanoma patients were compared to normal donors’ plasma samples. Using machine learning, we identified a 9 cfmiR signature that was associated with stage IV melanoma patients being non-responsive to CII. These cfmiRs were compared in pre- and post-treatment plasma samples from stage IV melanoma patients that showed good responses. Circulating miR-4649-3p, miR-615-3p, and miR-1234-3p demonstrated potential prognostic utility in assessing CII responses. Compared to LDH levels during CII, circulating miR-615-3p levels were consistently more efficient in detecting melanoma patients undergoing CII who developed progressive disease. By combining stage III/IV patients, 92 and 17 differentially expressed cfmiRs were identified in pre-treatment plasma samples from responder and non-responder patients, respectively. In conclusion, this pilot study demonstrated cfmiRs that identified treatment responses and could allow for real-time monitoring of patients receiving CII.

## 1. Introduction

Over the past decade, checkpoint inhibitor immunotherapy (CII) has significantly improved the outcomes of metastatic melanoma patients [1]. The CII monoclonal antibodies approved to treat metastatic melanoma patients include ipilimumab (targeting cytotoxic T lymphocyte-associated antigen 4, CTLA-4) [2], nivolumab and pembrolizumab (targeting programmed cell death protein-1, PD-1) [3]. Ipilimumab, nivolumab, and pembrolizumab represent the standard of care and are the most commonly utilized CII for treating metastatic melanoma patients [4]. One of the advantages of specific CII regimens is the durable response observed in melanoma patients even after treatment discontinuation, which can vary depending on the individual or combinatory CII implemented. Unfortunately, the complete response (CR) rate in melanoma patients is about 12–15% [5,6]. Major limitations for CIIs are primary and acquired CII resistance. Another limitation is the development of severe immune-related adverse events (IRAE), which forces the oncologist to discontinue the patient’s treatment [7]. Different tumor responses, tumor microenvironment changes, and host systemic immune responses play interactive roles in CII resistance, and IRAE [7,8]. Unfortunately, no key consistent findings and biomarkers have been found to identify these induced CII events earlier on patients.

Lactate dehydrogenase (LDH) is an enzyme involved in glucose metabolism that is highly expressed in rapidly growing tumors [9,10]. Due to the high energy demand from the tumor cells, glycolysis shifts from aerobic to anaerobic in a process called the Warburg effect [9]. Consequently, LDH expression increases in the cytosol of tumor cells, but in general will only reach the blood stream when the damaged cells release LDH [9]. Several prognostic blood biomarkers have been proposed for melanoma, but only serum LDH has been accepted as a prognostic biomarker for stage IV metastatic melanoma by the American Joint Committee on Cancer (AJCC) [11]. Therefore, the prognostic value of LDH has been assessed in metastatic melanoma patients receiving CII. In a prospective study, LDH and S100B have both been shown to be indicators of disease progression, although S100B was shown to be a better predictor of the development of distant metastasis [12]. Nevertheless, both failed at identifying high-risk patients with loco-regional metastasis and low tumor burden [12]. Elevated baseline LDH is an independent prognostic factor for overall survival (OS) in melanoma patients receiving ipilimumab [13], pembrolizumab, or ipilimumab and nivolumab combined [14]. Moreover, among different prognostic factors (LDH, tumor size, tumor PD-L1 status, ECOG performance status, *BRAF* mutation status, prior *BRAF* inhibitor targeted therapy, prior line of therapies, size of metastasis, and albumin levels), only low LDH baseline levels were associated with a CR to pembrolizumab [5]. Additionally, elevated LDH baseline levels were reduced at the first scan in melanoma patients receiving nivolumab or pembrolizumab, who had a better objective rate response when compared to patients with progressive disease (PD) [15]. To summarize, baseline LDH is a strong prognostic blood biomarker for stage IV melanoma patients, but has limitations. However, serum LDH assessment does not have informative utility for assessing stage III melanoma patients receiving CII.

Blood biomarkers are necessary for real-time monitoring of metastatic melanoma patients during treatment to allow for more effective decision making on treatment strategies. In the past several years, our group and others have shown that circulating cell-free nucleic acids (cfNA) have utility in monitoring metastatic melanoma patients undergoing treatment, particularly using circulating cell-free DNA (cfDNA) and circulating tumor cells (CTCs) [16,17,18,19,20,21,22,23]. The limitations of studying cfDNA in melanoma blood samples are the poor extraction efficacy from plasma, large volume of plasma required for assays, and the limited frequency of genomic aberrations in specific genes that are detectable [24,25]. The limitations in monitoring CTCs are the robustness of the isolation method used and the heterogeneity of the CTCs that can limit the interpretation of the findings. To find robust blood molecular biomarkers, our group has also focused on finding microRNAs (miRs) in melanoma patients’ blood [26] and tumor tissues [27,28]. MiRs are short sequence nucleic acids of 18–22 base pairs length that have a longer half-life and degrade minimally compared to cfDNA [29,30]. MiRs play significant roles in controlling and regulating mRNA expression, and thus lead to the activation/deactivation of specific molecular pathways [29,30]. In most of cancers, including melanoma, miRs are aberrantly expressed which affects molecular pathways controlling different cellular processes. These miRs can also be referred to as oncomiRs as they promote tumor development and progression. In melanoma several miRs have been proposed as tumor biomarkers to determine disease progression [29,31]. Also, significant efforts have been made in determining circulating cell-free miRs (cfmiRs) and exosomal miRs [29,31]. Recently, by using HTG EdgeSeq miR WTA, we found cfmiR signatures in plasma samples from patients with melanoma brain metastasis (MBM) [32]. Furthermore, we unraveled common cfmiR signatures in pre-operative plasma samples taken from stage III and IV melanoma patients receiving CIIs [32]. The advantage of using HTG EdgeSeq miR WTA to study cfmiRs is that we can directly profile and quantify >2000 miRs found in plasma samples by next-generation sequencing (NGS) to identify signature patterns [32]. Moreover, compared to other cfNA assays, the assay requires a minimal amount of plasma and no tedious extraction procedures.

Our hypothesis is that specific cfmiR signatures found in metastatic melanoma patients’ plasma samples allows us to perform multiple assessments and provides the clinician with the opportunity to monitor CII response in real-time. This is important in metastatic melanoma patients’ treatment management, as resistance to CII followed by rapid disease progression requires immediate decisions in order to prolong survival. In this study, we compared cfmiR expression to the standard blood protein biomarker LDH in stage IV melanoma patients. To carry this out, we screened for specific cfmiRs that were indicative of metastatic melanoma disease in pre- and post-treatment plasma samples from stage IV melanoma patients compared to normal donors’ plasma samples. By using machine learning we identified cfmiR signatures that were associated with CII response in stage IV responder and non-responder patients. Then, we compared the utility of these cfmiRs in predicting CII response in comparison to LDH levels at baseline and throughout the patients’ follow-ups. CfmiRs produced consistent results in predicting CII responses compared to elevated LDH levels at baseline and in longitudinal clinical assessment in stage IV melanoma patients. Finally, we identified cfmiRs that have potential in determining CII responses in both stage III and IV melanoma patients.

## 2. Results

### 2.1. LDH Levels at Treatment Baseline as a Predictive Factor for CII Response in Metastatic Melanoma Patients

**Figure 1:**
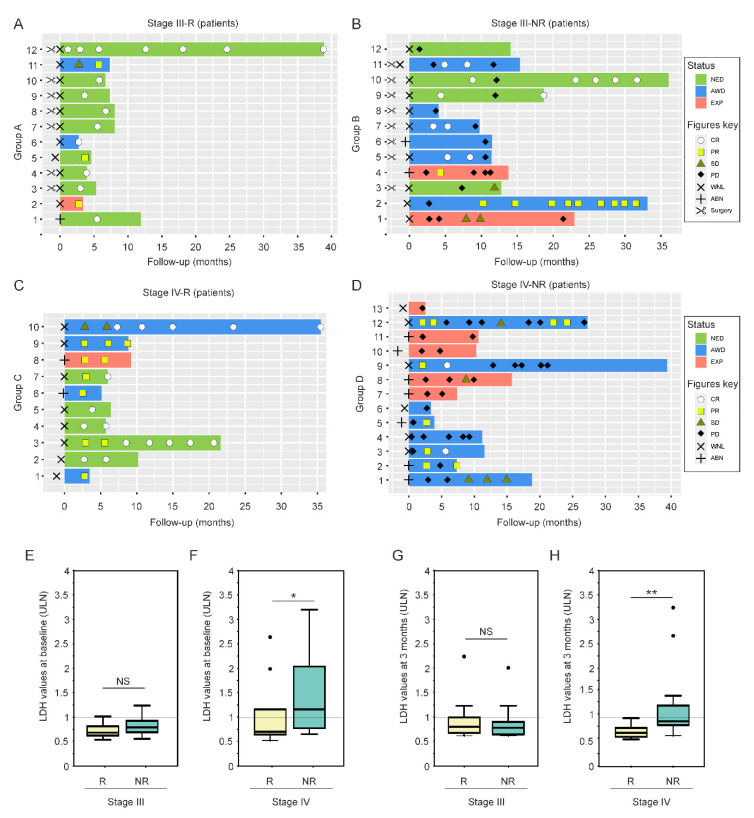
Melanoma patients and LDH assessment. (**A**,**B**) Swimmer plots showing disease status (NED, no evidence of disease; AWD, alive with disease; EXP, expired), RECIST 1.1 criteria (CR, complete response; PR, partial response; SD, stable disease; PD, progressive disease), surgery, and LDH levels (WNL, within normal; ABN, above normal) in stage III responder (Group A) (**A**) and non-responder (Group B) (**B**) melanoma patients. (**C**,**D**) Swimmer plots showing (NED, no evidence of disease; AWD, alive with disease; EXP, expired), RECIST 1.1 criteria (CR, complete response; PR, partial response; SD, stable disease; PD, progressive disease), surgery, and LDH levels (WNL, within normal; ABN, above normal) in stage IV responder (Group C) (**C**) and non-responder (Group D) (**D**) melanoma patients. (**E**) Boxplot showing the LDH values (ULN, upper limit normal) at baseline in stage III responder and non-responder melanoma patients (NS, non-significant). (**F**) Boxplot showing the LDH values (ULN) at baseline in stage IV responder and non-responder melanoma patients (* *p* < 0.05). (**G**) Boxplot showing the LDH values (ULN) at three months follow-up in stage III responder and non-responder melanoma patients (NS, non-significant). (**H**) Boxplot showing the LDH values (ULN) at three months follow-up in stage IV responder and non-responder melanoma patients (** *p* < 0.01). Dots represent outliers in each condition.

In order to identify cfmiRs associated with metastatic melanoma, we assessed plasma from a cohort of 47 melanoma patients (AJCC 8th edition stage III (n = 24) and IV (n = 23)) seen at the JWCI/SJHC clinic (Table 1). For each patient a range of 3–6 blood samples were collected and the samples were categorized as pre- or post-treatment according to the CII start date. Only plasma samples were included in the study and from this point on all the samples will be referred to as plasma. The samples were all analyzed using the HTG EdgeSeq miR WTA [32]. All of the patients analyzed had a median follow-up of 9.7 months and received CII (ipilimumab, nivolumab, pembrolizumab, or the combination of ipilimumab and nivolumab) as first line treatment. The 47 patients were divided into four different cohorts based on stage (III and IV) and CII response (responders and non-responders), which were analyzed by Response Evaluation Criteria In Solid Tumors (RECIST) 1.1 (Figure 1A–D). The four groups were as follows: stage III responder (group A); stage III non-responder (group B); stage IV responder (group C); and stage IV non-responder (group D). All of the patients had an LDH assessment taken at baseline and on longitudinal LDH assessments (average of 11 samples per patient) during CII (Figure 1A–D). LDH was considered elevated if patients had values taken >1 the upper limit normal (ULN) [23] (Table 1). Since LDH values at baseline were shown to be predictive of CII response in previous clinical studies [12,13,14,15], we initially compared the LDH levels at baseline for stage III and IV responder and non-responder patients. Although the sample size for this analysis is limited, the results showed a significantly higher expression of LDH levels at baseline in the stage IV non-responder group D when compared to the stage IV responder patients group C (Figure 1F). As expected, no differences were observed in responder and non-responder stage III patients (Figure 1E). Similar results were observed when the LDH values were assessed at 3 months after CII in both groups C and D (Figure 1G,H). These results are in agreement with previous observations showing that the LDH baseline levels predicts response in stage IV patients undergoing CII [15]. However, when assessing individual patients, the LDH levels were not of prognostic utility, since only ~54% of stage IV patients (7 of 13 patients) showed a correlation between high LDH levels and positive CII response. Importantly, the LDH values did not offer any advantage for stage III melanoma patients in relation to their response to CII.

### 2.2. Identification of cfmiRs in Pre- and Post-Treatment Samples from Patients Non-Responsive to CII

**Figure 2:**
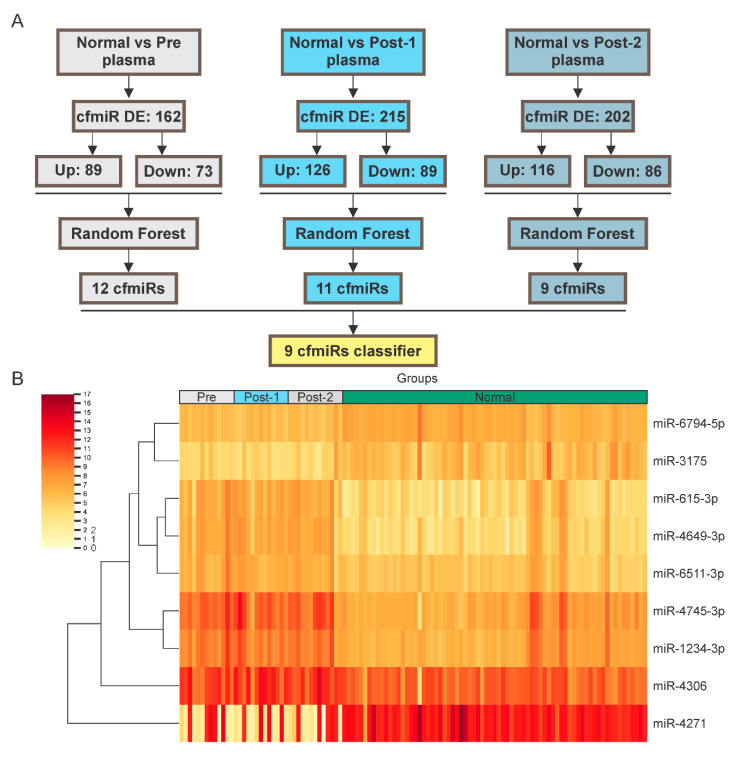
Identification of DE cfmiRs in normal donors’ and melanoma patients’ plasma samples (**A**) Shown are the DE cfmiRs in normal plasma samples versus pre- and post-treatment (Post-1 and Post-2) plasma samples. DE cfmiRs in each comparison were analyzed by the Random Forest algorithm. Specific classifiers were obtained for each analysis. A nine cfmiR classifier was commonly identified in all groups. (**B**) Heatmap showing the nine DE cfmiRs that were commonly identified in pre- and post-treatment plasma samples from stage IV non-responder melanoma patients. Scale bar showing the Log2 of normalized counts (ncounts).

In evaluating the utility of cfmiRs, it is important to find cfmiRs that have applicability in real-time monitoring of melanoma patient’s disease status before and during CII(s) to evaluate response. Recently, we have shown that specific cfmiR patterns found in MBM patients’ plasma may have utility in monitoring melanoma patients undergoing treatment [32]. Our hypothesis is that specific cfmiRs have a better utility compared to serum LDH levels in the assessment of melanoma patients undergoing CII. To address this hypothesis, pre-treatment samples (n = 13) from 13 stage IV melanoma patients who progressed (group D) were compared to normal donors’ samples (n = 73). A total of 162 differentially expressed (DE) cfmiRs were observed in the melanoma samples, of which 89 were upregulated and 73 were downregulated. To determine which cfmiRs classify metastatic melanoma patients from normal donors’ samples, we implemented a Random Forest algorithm to the 162 DE cfmiRs identified. The analysis generated a cfmiR classifier signature consisting of 12 cfmiRs (Figure 2A, Figure S1, and Table 2). To identify DE cfmiRs associated with disease progression during CII, 26 post-treatment samples collected from 13 stage IV non-responder (group D) melanoma patients were compared to normal donors’ samples. In each analysis 215 and 202 DE cfmiRs were found. Random Forest algorithm was applied to the 215 and 202 DE cfmiRs identified (Figure 2A, Figures S2 and S3). The top and commonly identified nine cfmiRs were selected as potential cfmiR biomarkers to monitor disease progression on melanoma patients undergoing CII (Table 2 and Figure 2B). Then, the levels of those nine cfmiRs were compared in pre-treatment, post-treatment, and normal donors’ samples. Of the nine cfmiRs identified, eight (miR-1234-3p, miR-3175, miR-4271, miR-4649-3p, miR-4745-3p, miR-615-3p, miR-6511-3p, and miR-6794-5p) were further evaluated since they showed significant changes in pre- and post-treatment samples from stage IV non-responders (Figure S4A–I). To summarize, using 13 paired blood samples (13 pre- and 26 post-treatment samples) nine cfmiRs were found as a potential biomarker for stage IV non-responder (group D) melanoma patients. Only eight of the nine cfmiRs were significantly DE in melanoma patients’ compared to normal donors’ samples.

### 2.3. MiR-615-3p Correlates with Melanoma Response to CII

**Figure 3:**
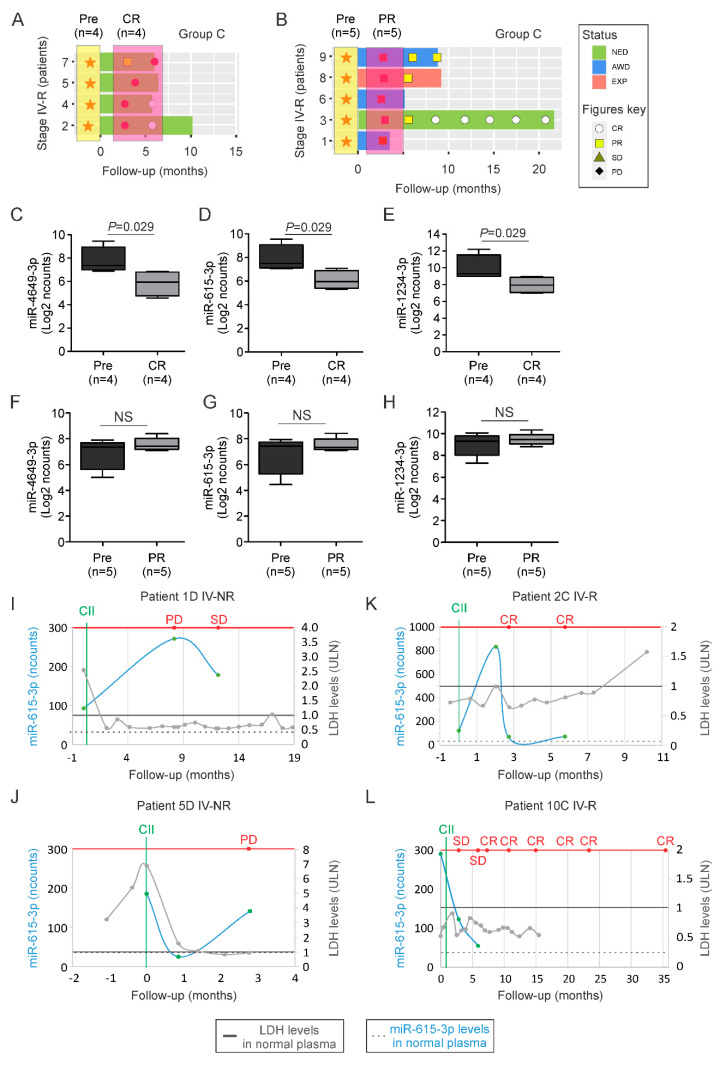
Validation of cfmiRs identified in assessing patient response to CIIs. (**A**,**B**) Disease status in stage IV patients who had a complete response (**A**, CR) or partial response (**B**, PR). Orange stars indicate pre-treatment (Pre) plasma samples. Red circles indicate blood collected in patients at CR. Red squares indicate blood collected in patients at PR. (**C**–**E**) Boxplots showing the changes in miR-4649-3p (**C**), miR-615-3p (**D**), and miR-1234-3p (**E**) levels in patients who achieved a CR (*p* values are indicated on top) compared to pre-treatment samples. (**F**–**H**) Boxplots showing the changes in miR-4649-3p (**F**) and miR-615-3p (**G**), and miR-1234-3p (**H**) levels in patients who achieved PR compared to pre-treatment samples (NS, non-significant). (**I**–**L**) Graph showing four melanoma patients: stage IV non-responder (IV-NR) patient 1D (**I**) or patient 5D (**J**); stage IV responders (IV-R) patient 2C (**K**) or patient 10C7 (**L**). Shown is the follow-up in months, LDH levels (labeled as light gray), and miR-615-3p levels (labeled as light blue; normalized counts, ncounts) at the indicated time points. Red line points to RECIST 1.1. Gray solid line indicates the upper limit normal (ULN) for LDH. Black dotted line indicates the average level of miR-615-3p detected in normal healthy donors’ plasma samples. Green solid line indicates the start of CII.

To determine whether the cfmiRs identified in stage IV non-responder patients had clinical utility to monitor patients’ treatment, we selected nine pre- and post-treatment samples from stage IV patients (group C) that responded to CII-treatment (achieved objective rate response, PR or CR). Of those nine patients, four reached CR (Figure 3A) and five patients had a partial response (PR) (Figure 3B). All of the samples were analyzed to determine the levels of the eight cfmiRs in the pre- and post-treatment samples. MiR-4649-3p, miR-1234-3p, and miR-615-3p levels significantly decreased in the post-treatment samples of the stage IV responder patients who had a CR (Figure 3C–E), but the levels did not change significantly for miR-3175, miR-4271, miR-4745-3p, miR-6511-3p, and miR-6794-5p (Figure S5A–E). On the contrary, in patients who had a PR no significant differences were observed in pre- and post-treatment samples for any of the eight cfmiRs assessed (Figure 3F–H and Figure S5F–J). To validate our observation, we assessed the expression levels of miR-615-3p in plasma samples from two stage IV responder and non-responder patients. Stage IV non-responder patients who progressed during CII had a significant increase in the expression of miR-615-3p (Figure 3I,J). In both cases LDH levels were unable to detect melanoma disease progression (Figure 3I,J). On the contrary, responder patients showed a decrease in miR-615-3p levels in post-treatment samples (Figure 3K,L). Similarly, LDH levels were also unable to detect CII response (Figure 3K,L). Then, we analyzed the detection levels of miR-615-3p for its ability to monitor stage III patients. To do that we compared pre- and post-treatment samples. The post-treatment samples were selected based on the patients’ RECIST 1.1 criteria. All of the patients had PD at some point during treatment, but at the time point of blood collection, only 8 patients had PD. MiR-651-3p was significantly increased in melanoma patients with PD compared to pre-treatment samples (Figure S6A). More importantly, miR-615-3p was able to monitor stage III non-responder melanoma patients during CII (Figure S6B). Finally, we compared the expression of miR-615-3p in pre- and post-treatment samples from stage III responders. For the 12 post-treatment samples selected, the patients achieved CR at the time point of blood collection. No significant differences were observed for miR-615-3p in stage III responder patients (Figure S6C). Similar analysis were performed for miR-4649-3p and the results were consistent with those observed for miR-615-3p (Figure S7A–E). To summarize, the cfmiR signature was successful in identifying stage IV responders during CII-treatment. MiR-4649-3p, miR-1234-3p, and miR-615-3p levels were associated with a CR in stage IV patients undergoing CIIs and were useful in monitoring responses of stage IV melanoma patients undergoing CII. Also, the results demonstrated differences for miR-615-3p in detecting stage III patients with PD, but failed to identify stage III patients with CR. 

### 2.4. A cfmiR Signature to Assess CII Responses in Stage III Melanoma Patients

**Figure 4:**
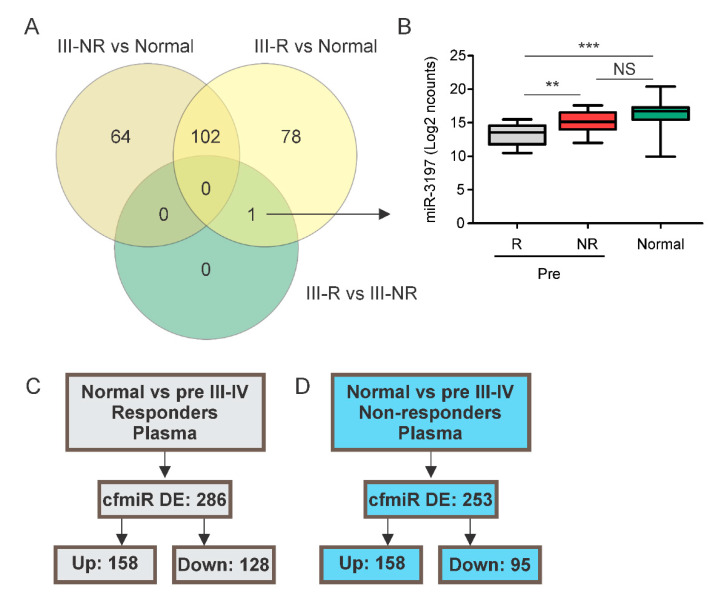
Characterization of cfmiRs in stage III melanoma patients. (**A**) Venn diagram showing the number of cfmiRs found in each comparison: stage III non-responders versus normal donors (III-NR vs. N); stage III responders versus normal donors (III-R vs. N); stage III responders versus non-responders (III-R vs. III-NR). (**B**) Boxplot showing the levels of miR-3197 in stage III responders (R), non-responders (NR), and normal donors’ plasma samples. (** *p* < 0.01, *** *p* < 0.001, NS, non-significant). (**C**) Shown are the DE cfmiRs in normal donors’ versus pre-treatment plasma samples from stage III/IV responder melanoma patients. (**D**) Shown are the DE cfmiRs in normal donors versus pre-treatment plasma samples in stage III/IV non-responders melanoma patients.

To find specific cfmiRs associated with stage III and CII response, 24 stage III patients (22 stage IIIC and 2 stage IIIB) undergoing CII were examined, of which 12 were responders (group A; Figure 1A) and 12 were non-responders (group B; Figure 1B). Initially, we compared the cfmiR expression in pre-treatment samples taken from stage III responders (group A) versus non-responders (group B). Surprisingly, miR-3197 was the only significantly DE cfmiR in the comparison (Figure 4A,B). MiR-3197 differentiated stage III responders from non-responders in pre-treatment samples (Figure 4B). Additionally, miR-3197 showed significant differences when comparing pre-treatment samples from responders versus normal donors’ samples (Figure 4B). This suggested that the cfmiRs detected in stage III patients are not significantly changing compared to normal donors’ samples. This is likely related to low tumor burden and low doubling time of stage III tumors being treated with CII.

In order to find biomarkers to monitor metastatic melanoma patients and increase our sample size, we combined stage III/IV melanoma patients and grouped them as non-responders and responders to CII. Then, non-responder and responder samples were compared to normal donors’ samples, respectively. A total of 286 DE cfmiRs (158 upregulated and 128 downregulated) were found in CII pre-treatment samples from the responder group compared to normal samples (Figure 4C). We then compared the pre-treatment samples from the non-responder patients versus normal donors’ samples. In the analysis, 253 DE cfmiRs (158 upregulated and 95 downregulated) were observed in non-responder patients compared to normal donors’ samples (Figure 4C). It is important to find cfmiRs that are useful for the monitoring of CII responses and to help distinguish metastatic melanoma responders from non-responders. Therefore, we focused on the detection of DE cfmiRs that were observed associated with non-response or response to CII. Therefore, we calculated the ratio of the FCs obtained in responders versus normal donors’ samples and in non-responders versus normal donors’ samples. Only cfmiRs with a ratio FC <0.75 were included. A total of 92 cfmiRs were DE in responders’ compared to normal donors’ and non-responders’ samples (Table S1).

We proposed that specific cfmiRs have the potential to identify patients who will respond to CII. By applying the same strategy but considering a ratio FC >1.25, 17 DE cfmiRs were found in stage III/IV non-responders’ compared to normal donors’ and responders’ samples (Table S2). The cfmiRs identified may represent potential biomarkers to determine patients who will likely develop PD to CII. MiR-1273e, miR-584-5p, and miR-1290 were found increased in non-responders stage III/IV melanoma patients. Surprisingly, the same cfmiRs were also found elevated in pre-operative MBM plasma samples as previously described by our group [32]. To summarize, 92 cfmiRs found in pre-treatment samples distinguished stage III/IV responders’ from non-responders’ and normal donors’ samples. On the contrary, 17 cfmiRs differentiated stage III/IV non-responders from responders and normal donors’ samples.

## 3. Discussion

Notwithstanding the large number of clinical and translational research studies, there is still a dire need for more reliable and informative blood biomarkers to better evaluate CII responses in real-time in melanoma patients. Metastatic melanoma progression can be rapid once tumors develop resistance to CIIs and bypass the host systemic immune control. Better blood biomarkers that can identify real-time changes in the patient’s disease status and allow for active monitoring could translate into earlier treatment decision making. There is evidence showing that higher baseline LDH values are associated with CII responses [13,14,15,33] and can allow for monitoring CII [15], but often the levels of LDH do not correlate with disease progression in patients receiving CII. Thus, it is difficult to rely on longitudinal LDH level assessment to make early clinical decisions in patients who are undergoing unsuccessful CII. Our study provides a detailed profiling of cfmiRs that potentially allow for the monitoring of stage III and IV melanoma patients during CII.

Despite the significant advances in improving progression-free survival (PFS) and OS, a high percentage of patients will still develop resistance and experience recurrence within the first year of starting CII [34]. Several studies have been conducted to identify miR biomarkers in melanoma tissues and/or plasma/serum that could predict melanoma progression [35,36]. However, most of the proposed cfmiRs are not validated or they represent single cfmiRs with limited reproducibility, and non-specific overlapping with benign diseases or normal healthy donor levels. In identifying biomarkers for CII response, some groups have focused on specific deregulated miRs in the tumor that can modulate the immune response against melanoma tumors, and thus control CII response. For example, miR-30b is upregulated in melanoma patients’ tissues and correlates to different clinical variables such as stage, metastatic potential, and shorter OS. MiR-30b promotes immunosuppression by targeting *GALNT7* (N-Acetylgalactosaminyltransferase 7) and increasing IL-10 production [37]. We observed an increased level of circulating cfmiR-30b in both responder and non-responder melanoma stage III/IV patients compared to normal donors’ samples. Also, an increase in cfmiR-30b levels was observed in responder compared to non-responder patients. In another study, miR-210 was shown to be upregulated in hypoxic areas of the tumor controlling cytotoxic T lymphocytes meditated lysis [38]. To summarize, miR-210 mediates its effects by targeting *PTPN1*, *HOXA1*, and *TP53I11* [38]. These studies support the role of elevated miRs in promoting melanoma progression in response to CII. However, the translational value of these findings into clinical biomarkers would require an assessment of the miRs in longitudinal biopsies of the tumor, which is not always feasible.

Blood biomarkers represent the most logistical and promising way to actively monitor patients in real-time during CII. Other studies have shown an eight cfmiR signature (miR-146a, miR-155, miR-125b, miR-100, let-7e, miR-125a, miR-146b, and miR-99b) found in extracellular vesicles released by metastatic melanoma tumors which were found to be associated with an increase in myeloid-derived suppressor cells and resistance to ipilimumab and nivolumab therapy [39]; however, not all of the cfmiRs identified were DE in melanoma patients when compared to normal donors’ samples. Our findings revealed that cfmiRs (miR-1234-3p, miR-3175, miR-4271, miR-4649-3p, miR-4745-3p, miR-615-3p, miR-6511-3p, and miR-6794-5p) are detected in pre-treatment plasma samples. Only miR-1234-3p, miR-4649-3p and miR-615-3p were significantly enhanced in post-treatment samples from stage IV non-responder patients. Accordantly, miR-4649-3p, miR-1234-3p, and miR-615-3p decreased in post-treatment samples of stage IV responder patients who had a CR during CII. Whereas, no significant differences were observed in stage IV responder patients who had a PR in comparison to pre-treatment samples. On longitudinal blood assessment, miR-615-3p and miR-4649-3p showed promising clinical utility in monitoring CII response in stage IV responder and non-responder patients. MiR-615-3p was previously detected and listed as a potential cfmiR for metastatic melanoma [40], but its function in melanoma is unknown [41]. To our knowledge, there is not report of the miR-4649-3p function in melanoma; however, it was previously reported that miR-4649-3p inhibits cell proliferation by targeting protein tyrosine phosphatase SHP-1 in nasopharyngeal carcinoma cells [42].

Identifying informative cfmiR biomarkers for stage IIIB-D melanoma is challenging, as the tumor size is variable ranging from multinodal micrometastasis to macrometastasis, with often variable growth rates. In this study, plasma samples derived from 24 melanoma patients undergoing CII (22 stage IIIC and 2 stage IIIB) were examined. As shown in previous studies [13,14,15,33] and in the present study, LDH baseline level assessment was successful in identifying most of stage IV patients, but it was not a reliable prognostic factor for stage III patients. When comparing stage III responders versus non-responders, only miR-3197 was found DE. Factors influencing the detection of cfmiRs changing could be associated with individual cfmiR variability, tumor burden, and tumor heterogeneity. To address this problem and to identify cfmiRs able to monitor metastatic melanoma, stage III and IV melanoma responders and non-responders were compared to normal donor samples. We found 92 cfmiRs associated with CII response. Whether any of these cfmiRs can be used as a robust biomarker will require further investigation. Also, 17 cfmiRs have shown potential applicability to determine stage III/IV melanoma patients who will not respond to CII. Relevant to this, we observed that miR-1273e, miR-584-5p, and miR-1290 have also been detected in MBM patients’ plasma. These cfmiRs may be indicators of III/IV melanoma patients who will eventually develop MBM. Recently, Walbrecq et al. identified miR-1290 as a novel hypoxia-associated miR, which is highly abundant in hypoxic extracellular vesicles released by melanoma cells [43].

Similar to CII resistance, different mechanisms have been associated with BRAF and MEK inhibitors resistance in metastatic melanoma. Often, these mechanisms over-activate the mitogen-activated protein kinase (MAPK) pathway and overcome BRAF and MEK inhibitors effects [44,45,46]. It has been proposed that similar mechanisms may drive CII resistance, as MAPK pathway can be over-active in CII-treated melanoma tumors [45]. Thus, it is also important to determine whether elevated miRs regulate MAPK pathway. In a previous study, high levels of miR-125b-5p were shown to be associated with Vemurafenib (BRAF inhibitor) resistance [47,48]. Accordingly, we observed that miR-125b was elevated in CII non-responder patients, but decreased in responding patients to CII (Table S1). Thus, miR-125b-5p may represent an example of overlapping roles for miRs in promoting cross-resistance to both BRAF and MEK inhibitors and CII.

We understand the limitations of our study in analyzing melanoma patients receiving different types of CII. Therefore, these findings may represent cfmiRs associated with responses to different CII. Future analyses are required to confirm and validate whether the cfmiRs have the ability to determine treatment response in well-defined cohort of patients receiving specific CII. To the best of our knowledge, this is the first report showing the potential ability of cfmiRs to distinguish patients who are non-responsive to CII from normal donors’ plasma samples. Further studies are needed to validate our observations in prospective clinical trials on larger sample sizes of metastatic melanoma patients undergoing CII(s).

## 4. Materials and Methods

### 4.1. Consent to Participate and Patient Specimen Accrual

This single-center study followed the principles found in the Declaration of Helsinki. All human samples and clinical information for this study were obtained according to the protocol guidelines approved by Saint John’s Health Center (SJHC)/John Wayne Cancer Institute (JWCI) Joint Institutional Review Board (IRB): JWCI Universal Consent (Providence Health and Services Portland IRB: JWCI-18-0401) and Western IRB: MORD-RTPCR-0995. Informed consent was obtained from all participants. The study was a prospective study designed to assess cfNA in CII-treated patients seen at JWCI/SJHC. All specimens were de-identified and entered into a restricted access database by a database operator.

### 4.2. Blood Sample Collection

Blood samples of healthy donors and melanoma patients were prospectively collected at SJHC/JWCI. Briefly, all blood samples were collected from 2016–2020 in Streck blood collection tubes (Streck, La Vista, NE, USA). Blood samples were accrued and processed to obtain plasma. Plasma was centrifuged, filtered, aliquoted, barcoded, and cryopreserved at −80°C as previously described [16]. Aliquots of plasma were thawed only once, mixed, and centrifuged before being analyzed by HTG EdgeSeq miR WTA.

For HTG EdgeSeq miR WTA analysis plasma samples (n = 73) were collected from normal healthy donors ranging in age from 21–65 years old, of which 41 were females and 32 were males. Pre-treatment samples (n = 47) were collected from AJCC 8th stage III and IV melanoma patients who received CII (Table 1). From the same CII- treated patients 2–5 blood samples (n = 111) that were collected post-treatment (after the first dose and during CII). All plasma samples were analyzed by HTG EdgeSeq miR WTA analysis. Overall, 158 melanoma plasma samples were analyzed from 47 patients. The melanoma patients analyzed had detailed clinical follow-up information and treatment response assessments as described in Section 4.3 below. The clinical demographics information for the 47 melanoma patients analyzed is summarized in Table 1.

### 4.3. CII Response

Every patient had a follow-up evaluation at the JWCI/SJHC cancer clinic as recommended in the current standard of care. The median follow-up was 9.7 months for the 47 patients analyzed. Each patient included in the study received at least four doses of the approved CII drugs (ipilimumab, nivolumab, pembrolizumab, or the combination of ipilimumab and nivolumab [16]) and were assessed for the RECIST 1.1. Briefly, CII responses were assessed using computerized tomography/magnetic resonance imaging every three months according to RECIST 1.1 criteria, denoting PD, SD (stable disease), PR, and CR. Based on RECIST 1.1 criteria the patients were stratified into responders (PR/CR) and non-responders (PD). Stage III patients who received surgery before receiving CII were considered NED until evaluated for RECIST 1.1. Stage III patients (3A, 4A, 7A, 8A, 9A, 10A, and 12A) from group A and patients (3B, 5B, 6B, 7B, 8B, 9B, 10B, and 11B) from group B received surgery and adjuvant treatment. This prospective study was performed in accordance with the REMARK guidelines [49,50].

LDH levels were evaluated using Dimension Vista LDH (LDI) Flex reagent cartridge (cat# K2054) an in vitro diagnostic test for the quantitative measurement of LDH in human serum on the Dimension Vista System analyzer (Siemens Medical Solutions Inc., PA, USA) at the SJHC Clinical Chemistry Department. LDH baseline and subsequent values were obtained for each patient. At least 3 LDH values were collected at different time points for all of the patients. Elevated LDH levels were considered in patients with >1X ULN (>240 U/L).

### 4.4. Sample Processing for HTG WTA

Melanoma patients’ and normal donors’ plasma samples were computer coded and de-identified during processing and assessing. The melanoma patients’ and normal donors’ plasma samples processing and NGS library preparation, quality control, normalized, and pooled was performed as described previously [32]. The pool library was sequenced on MiSeq or NextSeq 550 instruments following the respective Illumina instrument sequencing protocols. FASTQ files were generated from raw sequencing data using Illumina BaseSpace BCL to FASTQ software version 2.2.0 and Illumina Local Run Manager Software version 2.0.0. FASTQ files were analyzed with HTG EdgeSeq Parser software version v5.1.724.4793 to generate raw counts for 2083 miRs per sample. An .xls file containing the final counts for 2083 miRs per sample was generated for downstream data analysis. Data normalization was performed as discussed in Biostatistical analysis. Each HTG EdgeSeq miR WTA included negative (CTRL_ANT1, CTRL_ANT2, CTRL_ANT3, CTRL_ANT4, CTRL_ANT5) and positive (CTRL_miR_POS) miR controls. In all runs, Human Brain Total RNA (Ambion, Inc., Austin, TX, USA) was used as a control for library preparation, but they were not sequenced. All the samples that did not pass the quality control set by the HTG REVEAL software version 2.0.1 (Tuscon, AR, USA) were excluded from the analysis.

### 4.5. Biostatistical Analysis

The DESeq2 data normalization, analyses, and statistical comparisons for the melanoma (pre- and post-treatment) and normal donors’ plasma samples were all performed using the HTG REVEAL software version 2.0.1. In all of the comparison only cfmiRs with a log_2_ fold-change (Log_2_FC) >1.2 or <−1.2, a false discovery rate (FDR) > 0.05, and normalized counts greater than 30 were only considered. Ratio of the FCs was calculated by dividing the FC in non-responder versus normal to the FC of responder versus normal. Data normal distribution was evaluated by Shapiro-Wilk normality test. According to data normal distribution Kruskal-Wallis (non-normal distribution) tests were performed to determine differences among three or more groups. Mann-Whitney U-test (non-normal distribution) analysis was performed to compare differences between two groups. Box plots were performed with GraphPad Prism 5 (GraphPad Software Inc., La Jolla, CA, USA). To visualize the sequence and duration of treatments, patient response, and LDH levels, swimmer plots were employed using ggplot2 package version 3.3.2.9000 [51,52]. The swimmer plots were carried out using R version 4.0.0 (R Core Team) [52]. Data processing and Random Forest algorithm were performed using Python 3.7.7 using Scikit-learn and other packages as previously described in [32]. A two-sided *p*-value < 0.05 was considered statistically significant: * *p* < 0.05; ** *p* < 0.01; *** *p* < 0.001, and a *p*-value > 0.05 was considered non-significant (NS). The figures were processed using CorelDraw graphics suite 8X (Corel Corporation, Ottawa, Canada).

### 4.6. Data Deposit

The data generated and discussed in this study has been deposited in the NCBI’s Gene Expression Omnibus (GEO) and is accessible through the GEO Series accession number GSE157370.

## 5. Conclusions

In this prospective study, specific cfmiR signatures were found in plasma samples from metastatic melanoma patients. Three cfmiRs that were elevated in pre- and post-treatment plasma samples of stage IV non-responder patients were found to be downregulated in post-treatment plasma samples from patients who responded to CII and vice versa (see the Graphical Abstract). In addition, we proposed cfmiRs that may have the potential prognostic value to assess stage III/IV melanoma patients who will progress during CII. The present pilot study revealed specific cfmiRs that can help in monitoring CII response. MiR-615-3p and miR-4649-3p demonstrated a higher efficiency than LDH at baseline or during CII to monitor stage IV patients undergoing CII.

## Figures and Tables

**Table 1:** Clinical pathological information for metastatic melanoma patients receiving CII ^1^ analyzed for cfmiRs ^2^ in plasma samples.

	Melanoma Patients (n = 47)
Variables	n (%)
Age at diagnosis, mean (SD ^4^)	62.0 (13.9)
Age at treatment, mean (SD ^4^)	65.9 (13.5)
<60	14 (29.8)
≥60	33 (70.2)
Gender	
Male	30 (63.8)
Female	17 (36.2)
Treatment regimen	
Anti-PD-1	31 (65.95)
Anti-PD-1/anti-CTLA-4	16 (34.05)
AJCC 8th ed. Stages ^3^	
III b/c	24 (51.1)
IV a/b/c/d	23 (48.9)
*BRAF* mutation	
Positive	25 (53.2)
Negative	22 (46.8)
CII-response based on RECIST ^5^ 1.1	
Responders	22 (46.8)
Non-responders	25 (53.2)
Number of metastasis	
1	24 (51.06)
≥2	16 (34.04)
unknown	7 (14.90)
LDH ^6^ level at baseline	
≤1X ^7^ ULN	35 (74.5)
>1X ULN	12 (23.5)

^1^ CII = checkpoint immune inhibitor. ^2^ Cell-free microRNAs = cfmiRs. ^3^ AJCC 8th stage = American Joint Committee on Cancer 8th edition determined at the start date of CII. ^4^ SD = standard deviation. ^5^ RECIST = Respond Evaluation Criteria In Solid Tumors. ^6^ LDH = lactate dehydrogenase. ^7^ ULN = upper limit normal.

**Table 2:** CfmiR ^1^ classifiers commonly identified by Random Forest in stage IV patients that had PD ^2^.

Probe	Pre-Treatment FIS ^3^	Post-Treatment-1 FIS ^3^	Post-Treatment-2 FIS ^3^
**miR-4271**	0.06	0.05	0.07
**miR-3175**	0.05	0.05	0.04
**miR-4745-3p**	0.04	0.01	0.02
**miR-6862-3p**	0.03	N/A	N/A
**miR-4649-3p**	0.03	0.04	0.02
**miR-6510-3p**	0.02	0.01	N/A
**miR-4306**	0.01	0.02	0.02
**miR-1234-3p**	0.01	0.05	0.01
**miR-6511a-3p**	0.01	0.04	0.01
**miR-615-3p**	0.01	0.02	0.02
**miR-6794-5p**	0.01	0.02	0.03
**miR-1301-5p**	0.01	0.02	N/A

^1^ CfmiR = cell-free miRNA. ^2^ PD = progressive disease. ^3^ FIS = Feature Importance Scores.

## Supplementary Materials

The following are available online at https://www.mdpi.com/2072-6694/12/11/3361/s1, Figure S1: Random Forest algorithm in pre-treatment samples from stage IV non-responder patients, Figure S2: Random Forest algorithm in post-treatment-1 samples from stage IV non-responder patients, Figure S3: Random Forest algorithm in post-treatment-2 samples from stage IV non-responder patients, Figure S4: CfmiRs detection levels in pre- and post-treatment-1 and 2 samples from stage IV non-responder patients, Figure S5: CfmiR levels in pre- and post-CII-treated melanoma patients, Figure S6: MiR-615-3p levels in longitudinal bloods from stage III non-responder patients, Figure S7: MiR-4649-3p levels in longitudinal bloods from stage IV non-responder patients, Table S1: CfmiR identified in pre-treatment samples of stage III/IV responders, Table S2: CfmiR identified in pre-treatment samples of stage III/IV non-responders.

## Abbreviations

AJCC	American Joint Committee on Cancer
CfmiR	Cell-free miR
CfDNA	Cell-free DNA
CfNA	Cell-free Nucleic Acids
CII	Checkpoint Inhibitor Immunotherapy
CR	Complete Response
CTLA-4	Cytotoxic T Lymphocyte-Associated Antigen 4
IRAE	Immune-Related Adverse Events
DE	Differentially Expressed
FC	Fold-Change
FDR	False Discovery Rate
GEO	Gene Expression Omnibus
MBM	Melanoma Brain Metastasis
miR	MicroRNA
NED	No Evidence of Disease
NGS	Next-Generation Sequencing
PD-1	Programmed Cell Death Protein-1
PD-L1	Programmed Cell Death Protein-1 Ligand
PD	Progressive Disease
PFS	Progressive-Free Survival
PR	Partial Response
NS	Non-Significant
RECIST	Response Evaluation Criteria In Solid Tumors 1.1
REMARK	Reporting Recommendations For Tumour Marker
SD	Stable Disease
LDH	Lactate Dehydrogenase
ULN	Upper Limit Normal
WTA	Whole Transcriptome Assay
