# Supplementary material for: A Pilot Study Comparing the Efficacy of Lactate Dehydrogenase Levels Versus Circulating Cell-Free microRNAs in Monitoring Responses to Checkpoint Inhibitor Immunotherapy in Metastatic Melanoma Patients
Source: Cancers (Basel). 2020 Nov 13;12(11):3361. doi: 10.3390/cancers12113361 (PMC7696545; doi:10.3390/cancers12113361)
Supplement: Supplementary file 1 [file cancers-12-03361-s001.zip › cancers-1007326-XML suppl/cancers-1007326-XML suppl.docx]

Supplementary Materials

A Pilot Study Comparing the Efficacy of Lactate Dehydrogenase Levels versus Circulating Cell-Free microRNAs in Monitoring Responses to Checkpoint Inhibitor Immunotherapy in Metastatic
Melanoma Patients

Matias A. Bustos, Rebecca Gross, Negin Rahimzadeh, Hunter Cole, Linh T. Tran,
Kevin D. Tran, Ling Takeshima, Stacey L. Stern, Steven O’Day and Dave S. B. Hoon

Supplementary Figures

**Supplementary Figure 1.** Random Forest algorithm in pre-treatment samples from stage IV non-responder patients. Feature importance scores of cfmiRs in classifying normal versus pre-treatment samples from stage-IV non-responder patients from the Random Forest classifier.

**Supplementary Figure 2.** Random Forest algorithm in post-treatment-1 samples from stage IV non-responder patients. Feature importance scores of cfmiRs in classifying normal versus post-treatment (Post-1) samples from stage-IV non-responder patients from the Random Forest classifier.

**Supplementary Figure 3.** Random Forest algorithm in post-treatment-2 samples from stage IV non-responder patients. Feature importance scores of cfmiRs in classifying normal versus post-treatment (Post-2) samples from stage-IV non-responder patients from the Random Forest classifier.

**Supplementary Figure 4.** CfmiRs detection levels in pre- and post-treatment-1 and 2 samples from stage IV non-responder patients. (**A**–**I**) Boxplots showing the level of miR-4649-3p (**A**), miR-615-3p (**B**), miR-6511-3p (**C**), miR-6794-5p (**D**), miR-1234-3p (**E**), miR-3175 (**F**)**,** miR-4271 (**G**), miR-4306 (**H**)**,** and miR-4745 (**I**) in pre-treatment (Pre), post-treatment (Post-1 and Post-2), and normal plasma samples from stage IV non-responder patients (NS, non-significant; * *P* < 0.05, ** *P* < 0.01, *** *P* < 0.001).

**Supplementary Figure 5.** CfmiR levels in pre- and post-CII treated melanoma patients. (**A**–**E**) Boxplot showing the level of miR-4745 (**A**), miR-6511-3p (**B**), miR-6794-5p (**C**), miR-4271 (**D**), miR-3175 (**E)** in patients who achieved a CR (NS, non-significant). (**F**–**J**) Boxplot showing the level of miR-4745-3p (**F**), miR-6511-3p (**G**), miR-6794-5p (**H**), miR-4271 (**I**), miR-3175 (**J**) in patients who achieved a PR (NS, non-significant).

**Supplementary Figure 6.** MiR-615-3p levels in longitudinal bloods from stage III non-responder patients. (**A**) Boxplots showing the changes in miR-615-3p levels stage III patient non-responders when patients developed progressive disease (PD, *P* values are indicated on top) compared to pre-treatment samples. (**B**) Graph showing four melanoma patients: stage III non-responder (III-NR) patient 1B. Shown is the follow-up in months, LDH levels (labeled as light gray), and miR-615-3p levels (labeled as light blue; normalized counts, ncounts) at the indicated time points. Red line points to RECIST 1.1. Gray solid line indicates the upper limit normal (ULN) for LDH. Black dotted line indicates the average level detected in normal plasma samples. Green solid line indicates the start of CII. (**C**) Boxplots showing the changes in miR-615-3p levels stage III patient responders when patients developed complete response (CR) compared to pre-treatment samples.

**Supplementary Figure 7.** MiR-4649-3p levels in longitudinal bloods from stage IV non-responder patients. (**A**–**E**) Graphs showing four melanoma patients: stage IV non-responder (IV-NR) patient 1D (**A**) or patient 5D (**B**); stage IV responders (IV-R) patient 2C (**C**) or patient 10C (**D**); stage III non-responder (III-NR) patient 1B (**E**). Shown is the follow-up in months, LDH levels (labeled as light gray), and miR-4649-3p levels (labeled as light blue; normalized counts, ncounts) at the indicated time points. Red line points to RECIST 1.1. Gray solid line indicates the upper limit normal (ULN) for LDH. Black dotted line indicates the average level detected in normal plasma samples. Green solid line indicates the start of CII.

**Table S1.** ^1^CfmiR identified in pre-treatment samples of stage III/IV responders.

| **Probe** | ^2^**FC** ^3^**NR vs** ^4^**N** | ^2^**FC** ^5^**R vs** ^4^**N** | **Adjusted P** ^3^**NR vs** ^4^**N** | **Adjusted P** ^5^**R vs** ^4^**N** | **Ratio of** ^2^**FC** |
| --- | --- | --- | --- | --- | --- |
| **miR-144-3p** | 2.09 | 4.66 | 1.50E-03 | 4.96E-10 | 0.45 |
| **miR-144-5p** | 2.51 | 5.36 | 8.22E-05 | 5.15E-12 | 0.47 |
| **miR-122-5p** | -1.62 | -3.26 | 2.84E-02 | 5.51E-07 | 0.50 |
| **miR-3197** | -2.08 | -3.96 | 1.50E-03 | 2.08E-08 | 0.53 |
| **miR-96-5p** | 2.64 | 5.00 | 1.75E-05 | 5.91E-12 | 0.53 |
| **miR-32-5p** | 1.57 | 2.97 | 1.67E-02 | 5.98E-08 | 0.53 |
| **miR-6782-5p** | 1.67 | 3.03 | 1.11E-02 | 3.79E-07 | 0.55 |
| **miR-127-3p** | -1.10 | -1.91 | 7.06E-01 | 1.21E-02 | 0.58 |
| **miR-4293** | 1.18 | 2.01 | 3.05E-01 | 3.94E-05 | 0.59 |
| **miR-6798-5p** | -1.18 | -2.00 | 4.32E-01 | 2.30E-03 | 0.59 |
| **miR-326** | -1.10 | -1.79 | 6.62E-01 | 7.90E-03 | 0.61 |
| **miR-101-3p** | 1.64 | 2.64 | 9.40E-03 | 3.04E-06 | 0.62 |
| **miR-382-5p** | 1.05 | -1.69 | 8.29E-01 | 3.31E-02 | 0.62 |
| **miR-4745-3p** | 3.47 | 5.56 | 5.68E-09 | 3.55E-15 | 0.62 |
| **miR-182-5p** | 2.70 | 4.32 | 1.84E-05 | 2.45E-09 | 0.63 |
| **miR-4530** | 1.90 | 3.04 | 4.15E-04 | 9.43E-09 | 0.63 |
| **miR-1234-3p** | 3.20 | 5.08 | 4.90E-09 | 1.30E-15 | 0.63 |
| **miR-4459** | -1.52 | -2.41 | 1.90E-02 | 5.66E-06 | 0.63 |
| **miR-142-5p** | 1.53 | 2.42 | 6.96E-02 | 5.43E-04 | 0.63 |
| **miR-183-5p** | 1.82 | 2.87 | 1.30E-03 | 1.31E-07 | 0.63 |
| **miR-6723-5p** | -1.29 | -2.02 | 3.08E-01 | 8.50E-03 | 0.64 |
| **miR-1208** | 1.41 | 2.20 | 4.91E-02 | 3.46E-05 | 0.64 |
| **miR-3940-5p** | -1.38 | -2.15 | 6.22E-02 | 4.42E-05 | 0.64 |
| **miR-6895-3p** | 1.66 | 2.56 | 7.01E-04 | 2.54E-09 | 0.65 |
| **miR-668-5p** | 1.47 | 2.25 | 3.15E-02 | 2.72E-05 | 0.65 |
| **miR-26b-5p** | 2.44 | 3.73 | 3.01E-04 | 5.55E-07 | 0.65 |
| **miR-4430** | -1.29 | -1.97 | 1.89E-01 | 1.20E-03 | 0.65 |
| **miR-16-5p** | 2.03 | 3.10 | 2.80E-03 | 1.10E-05 | 0.65 |
| **miR-6124** | -1.03 | -1.57 | 8.72E-01 | 2.22E-02 | 0.66 |
| **miR-6741-5p** | 1.45 | 2.19 | 3.30E-02 | 3.30E-05 | 0.66 |
| **miR-548d-5p** | -1.17 | -1.76 | 4.89E-01 | 2.07E-02 | 0.66 |
| **miR-6511a-3p** | 2.46 | 3.69 | 2.12E-07 | 2.36E-13 | 0.67 |
| **miR-6081** | -1.62 | -2.43 | 8.14E-02 | 3.00E-03 | 0.67 |
| **miR-615-3p** | 3.46 | 5.18 | 1.24E-08 | 2.24E-13 | 0.67 |
| **miR-1247-5p** | 1.57 | 2.35 | 1.70E-03 | 2.89E-08 | 0.67 |
| **miR-6789-3p** | 1.44 | 2.13 | 3.70E-03 | 2.35E-08 | 0.68 |
| **miR-15a-5p** | 1.87 | 2.76 | 7.30E-03 | 6.83E-05 | 0.68 |
| **miR-451a** | 1.34 | 1.97 | 1.70E-01 | 3.60E-03 | 0.68 |
| **miR-15b-5p** | 1.98 | 2.91 | 5.60E-03 | 6.91E-05 | 0.68 |
| **miR-193a-5p** | -1.05 | -1.54 | 7.01E-01 | 2.60E-03 | 0.68 |
| **miR-4304** | -1.00 | 1.46 | 9.80E-01 | 5.20E-03 | 0.68 |
| **miR-6126** | -1.42 | -2.07 | 4.06E-02 | 1.05E-04 | 0.69 |
| **miR-454-3p** | 2.36 | 3.44 | 1.76E-05 | 5.41E-09 | 0.69 |
| **miR-4292** | 1.97 | 2.87 | 2.43E-06 | 1.90E-12 | 0.69 |
| **miR-4722-3p** | 2.08 | 3.01 | 1.98E-06 | 5.15E-12 | 0.69 |
| **miR-6774-3p** | 1.68 | 2.41 | 1.46E-02 | 1.52E-04 | 0.70 |
| **let-7f-5p** | 2.39 | 3.42 | 8.02E-05 | 1.62E-07 | 0.70 |
| **miR-340-5p** | 1.68 | 2.40 | 1.18E-02 | 1.06E-04 | 0.70 |
| **miR-619-3p** | -1.54 | -2.19 | 1.40E-01 | 1.18E-02 | 0.70 |
| **miR-425-3p** | -1.28 | -1.82 | 2.32E-01 | 7.10E-03 | 0.70 |
| **miR-6127** | -1.38 | -1.96 | 7.07E-02 | 6.00E-04 | 0.70 |
| **miR-6877-5p** | -1.19 | -1.69 | 3.25E-01 | 7.10E-03 | 0.70 |
| **miR-5196-5p** | -1.12 | -1.59 | 4.84E-01 | 6.20E-03 | 0.70 |
| **miR-6510-3p** | 2.39 | 3.39 | 3.31E-07 | 4.13E-12 | 0.71 |
| **miR-6880-5p** | -1.03 | -1.46 | 8.17E-01 | 1.28E-02 | 0.71 |
| **miR-6781-5p** | -1.08 | -1.53 | 6.59E-01 | 2.05E-02 | 0.71 |
| **miR-328-3p** | -1.23 | -1.74 | 0.3401 | 0.0162 | 0.71 |
| **miR-7851-3p** | -1.16 | -1.64 | 0.3442 | 0.0049 | 0.71 |
| **miR-1539** | 1.04 | 1.47 | 0.8058 | 0.011 | 0.71 |
| **miR-194-5p** | 1.79 | 2.53 | 0.0029 | 1.26064E-05 | 0.71 |
| **miR-1285-5p** | -1.15 | -1.62 | 0.543 | 0.0459 | 0.71 |
| **miR-6894-5p** | -1.33 | -1.87 | 0.0898 | 0.000675148 | 0.71 |
| **miR-185-3p** | -1.38 | -1.94 | 0.1006 | 0.0018 | 0.71 |
| **miR-6869-5p** | 1.21 | 1.7 | 0.183 | 0.000503238 | 0.71 |
| **miR-29b-3p** | 1.71 | 2.4 | 0.0068 | 5.2978E-05 | 0.71 |
| **miR-3169** | -1.47 | -2.05 | 0.1755 | 0.0188 | 0.72 |
| **miR-6086** | -1.37 | -1.91 | 0.0493 | 0.000242847 | 0.72 |
| **miR-504-3p** | -1.22 | -1.7 | 0.2089 | 0.0021 | 0.72 |
| **miR-424-5p** | 1.17 | 1.63 | 0.3897 | 0.0096 | 0.72 |
| **miR-3689a-3p** | -2.24 | -3.12 | 0.0001656 | 1.00739E-06 | 0.72 |
| **miR-491-5p** | -1.1 | -1.53 | 0.6132 | 0.0388 | 0.72 |
| **miR-7109-3p** | 1.36 | 1.89 | 0.0175 | 7.12662E-06 | 0.72 |
| **miR-4726-3p** | 1.6 | 2.22 | 0.000438152 | 2.88796E-08 | 0.72 |
| **miR-4649-3p** | 3.7 | 5.11 | 3.75709E-10 | 1.13034E-14 | 0.72 |
| **miR-6727-5p** | -1.32 | -1.82 | 0.1266 | 0.0025 | 0.73 |
| **miR-125b-5p** | -1.06 | -1.46 | 0.7008 | 0.0129 | 0.73 |
| **miR-8071** | -1.57 | -2.16 | 0.0172 | 0.000244615 | 0.73 |
| **miR-4534** | -1.04 | -1.43 | 0.8016 | 0.0224 | 0.73 |
| **miR-935** | 1.78 | 2.44 | 0.000161816 | 4.27463E-08 | 0.73 |
| **miR-4478** | -1.19 | -1.63 | 0.2106 | 0.0011 | 0.73 |
| **miR-4319** | 1.59 | 2.17 | 0.000254794 | 9.42538E-09 | 0.73 |
| **miR-1273g-5p** | -1.24 | -1.69 | 0.177 | 0.0026 | 0.73 |
| **let-7g-5p** | 1.77 | 2.41 | 0.0078 | 0.000195765 | 0.73 |
| **miR-3141** | -1.5 | -2.04 | 0.0098 | 3.46264E-05 | 0.74 |
| **miR-6762-3p** | 1.25 | 1.7 | 0.1518 | 0.0018 | 0.74 |
| **miR-4315** | 1.2 | 1.62 | 0.2155 | 0.002 | 0.74 |
| **miR-6769a-3p** | 1.07 | 1.44 | 0.6093 | 0.0071 | 0.74 |
| **miR-6816-3p** | 2.66 | 3.56 | 1.62691E-05 | 1.31366E-07 | 0.74 |
| **miR-20b-5p** | 1.66 | 2.22 | 0.0102 | 0.000218285 | 0.74 |
| **miR-6720-5p** | 2.26 | 3.02 | 1.9888E-05 | 5.14405E-08 | 0.74 |
| **miR-4515** | 1.61 | 2.15 | 0.0032 | 1.59537E-05 | 0.74 |
| **miR-363-3p** | 1.73 | 2.31 | 0.0033 | 4.882E-05 | 0.74 |

^1^CfmiR = cell-free miRNA. ^2^FC= fold-change; ^3^NR=non-responders; ^4^N=normal; ^5^R= responders.

**Table S2.** ^1^CfmiR identified in pre-treatment samples of stage III/IV non-responders.

| **Probe** | ^2^**FC** ^3^**NR vs** ^4^**N** | ^2^**FC** ^5^**R vs** ^4^**N** | **Adjusted P** ^3^**NR vs** ^4^**N** | **Adjusted P** ^5^**R vs** ^4^**N** | **Ratio of** ^2^**FCs** |
| --- | --- | --- | --- | --- | --- |
| **miR-654-3p** | 2.03 | 1.59 | 6.90E-04 | 4.22E-02 | 1.28 |
| **miR-494-3p** | 1.99 | 1.42 | 8.72E-04 | 1.31E-01 | 1.40 |
| **miR-6131** | 1.87 | 1.32 | 6.43E-04 | 1.80E-01 | 1.42 |
| **miR-1301-5p** | -2.00 | -1.26 | 2.98E-05 | 2.08E-01 | 1.59 |
| **miR-584-5p** | 1.95 | 1.39 | 4.70E-03 | 2.19E-01 | 1.40 |
| **miR-136-5p** | 1.97 | 1.37 | 4.20E-03 | 2.39E-01 | 1.44 |
| **miR-370-3p** | 1.78 | 1.27 | 4.50E-03 | 3.09E-01 | 1.40 |
| **miR-1273e** | 1.59 | 1.23 | 1.05E-02 | 3.22E-01 | 1.29 |
| **miR-539-5p** | 1.56 | 1.23 | 3.12E-02 | 3.85E-01 | 1.27 |
| **miR-1290** | 2.00 | 1.19 | 1.67E-04 | 4.03E-01 | 1.68 |
| **miR-376c-3p** | 1.70 | 1.23 | 1.50E-02 | 4.04E-01 | 1.38 |
| **miR-432-5p** | 1.74 | 1.20 | 9.30E-03 | 4.60E-01 | 1.45 |
| **miR-487b-3p** | 1.54 | 1.20 | 4.04E-02 | 4.62E-01 | 1.28 |
| **miR-485-3p** | 1.67 | 1.16 | 8.80E-03 | 5.26E-01 | 1.44 |
| **miR-376a-3p** | 1.78 | 1.13 | 6.20E-03 | 6.28E-01 | 1.58 |
| **miR-143-3p** | 1.61 | 1.10 | 3.27E-02 | 7.31E-01 | 1.46 |
| **miR-377-3p** | 1.64 | 1.07 | 8.60E-03 | 7.79E-01 | 1.53 |

^1^CfmiR = cell-free miRNA. ^2^FC= fold-change; ^3^NR=non-responders; ^4^N=normal; ^5^R= responders.
