# Supplementary figures and images for: A Pilot Study Comparing the Efficacy of Lactate Dehydrogenase Levels Versus Circulating Cell-Free microRNAs in Monitoring Responses to Checkpoint Inhibitor Immunotherapy in Metastatic Melanoma Patients
Source: Cancers (Basel). 2020 Nov 13;12(11):3361. doi: 10.3390/cancers12113361 (PMC7696545; doi:10.3390/cancers12113361)

Figure S1

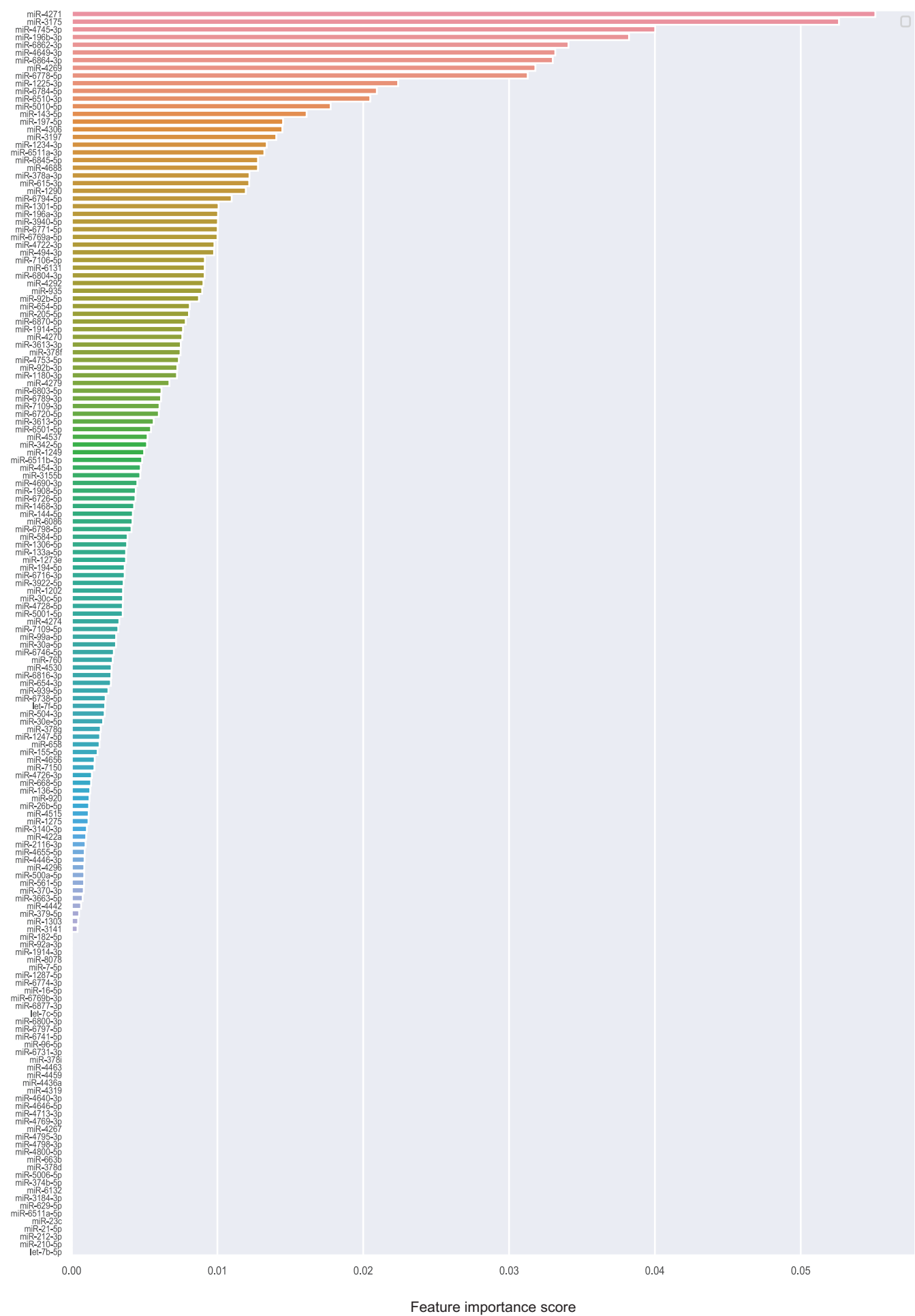

Supplement: Supplementary file 1 [file cancers-12-03361-s001.zip › cancers-1007326-XML suppl/Figure S1.pdf]

Figure S2

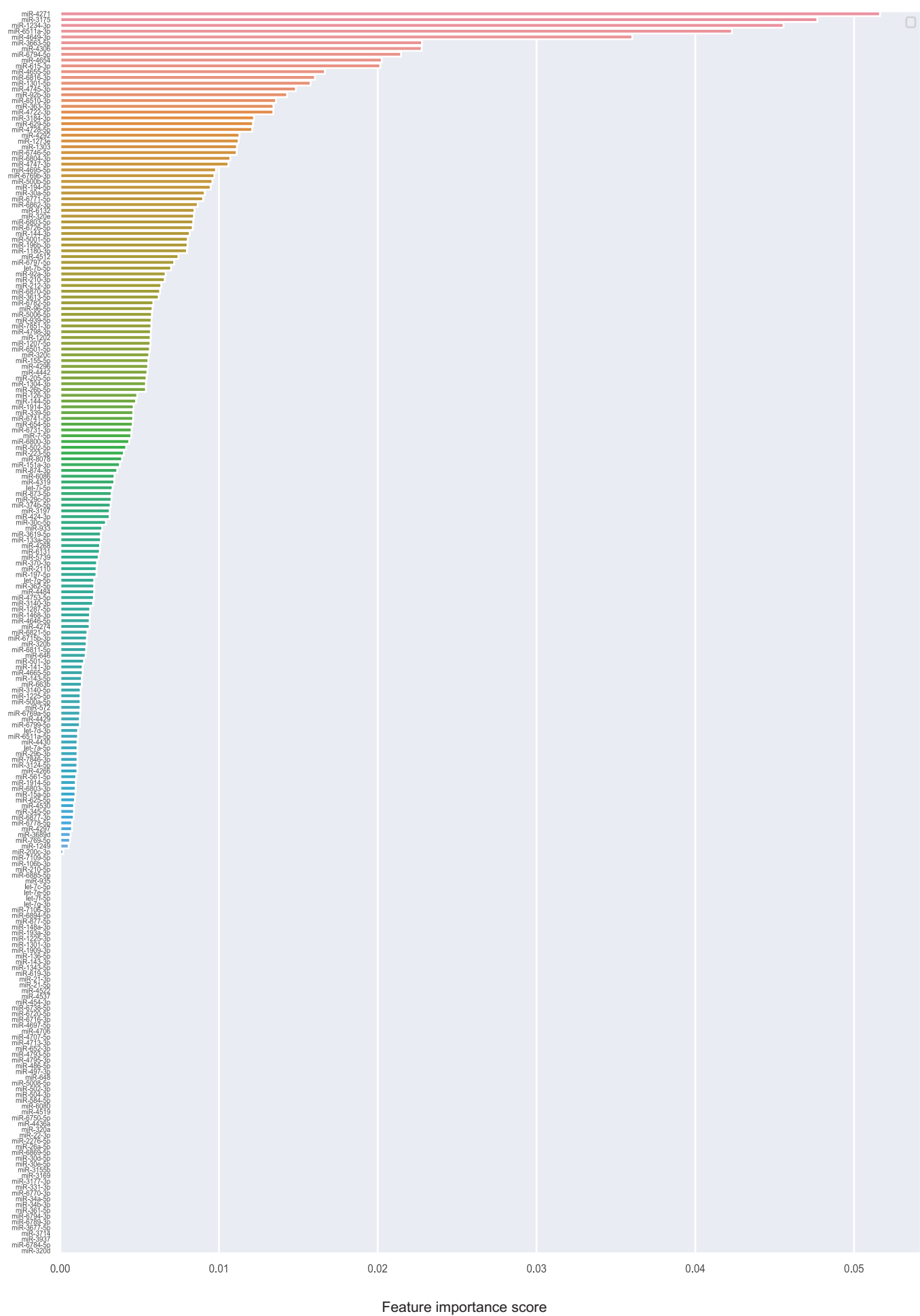

Supplement: Supplementary file 1 [file cancers-12-03361-s001.zip › cancers-1007326-XML suppl/Figure S2.pdf]

### Figure S3

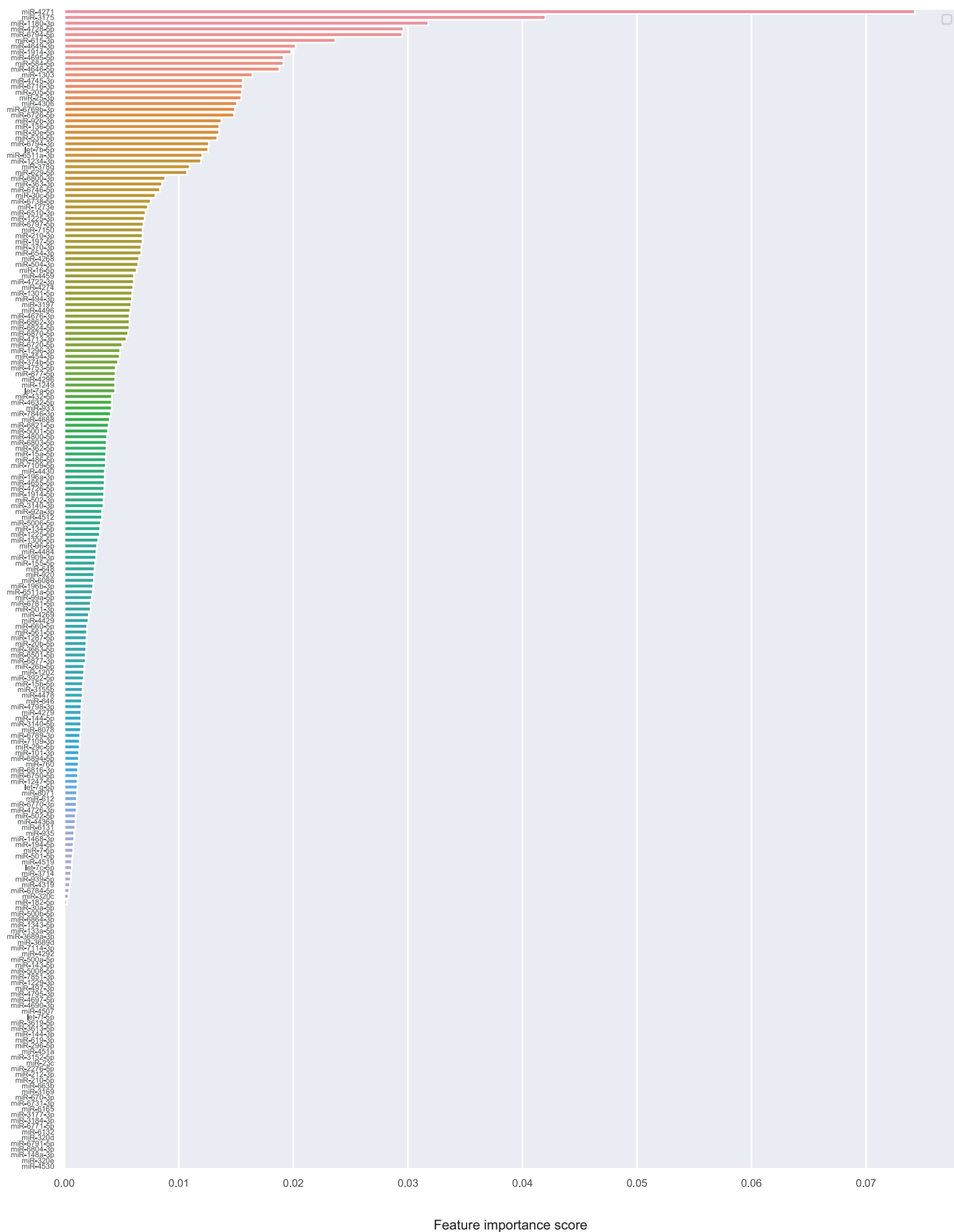

Supplement: Supplementary file 1 [file cancers-12-03361-s001.zip › cancers-1007326-XML suppl/Figure S3.pdf]

Figure S4

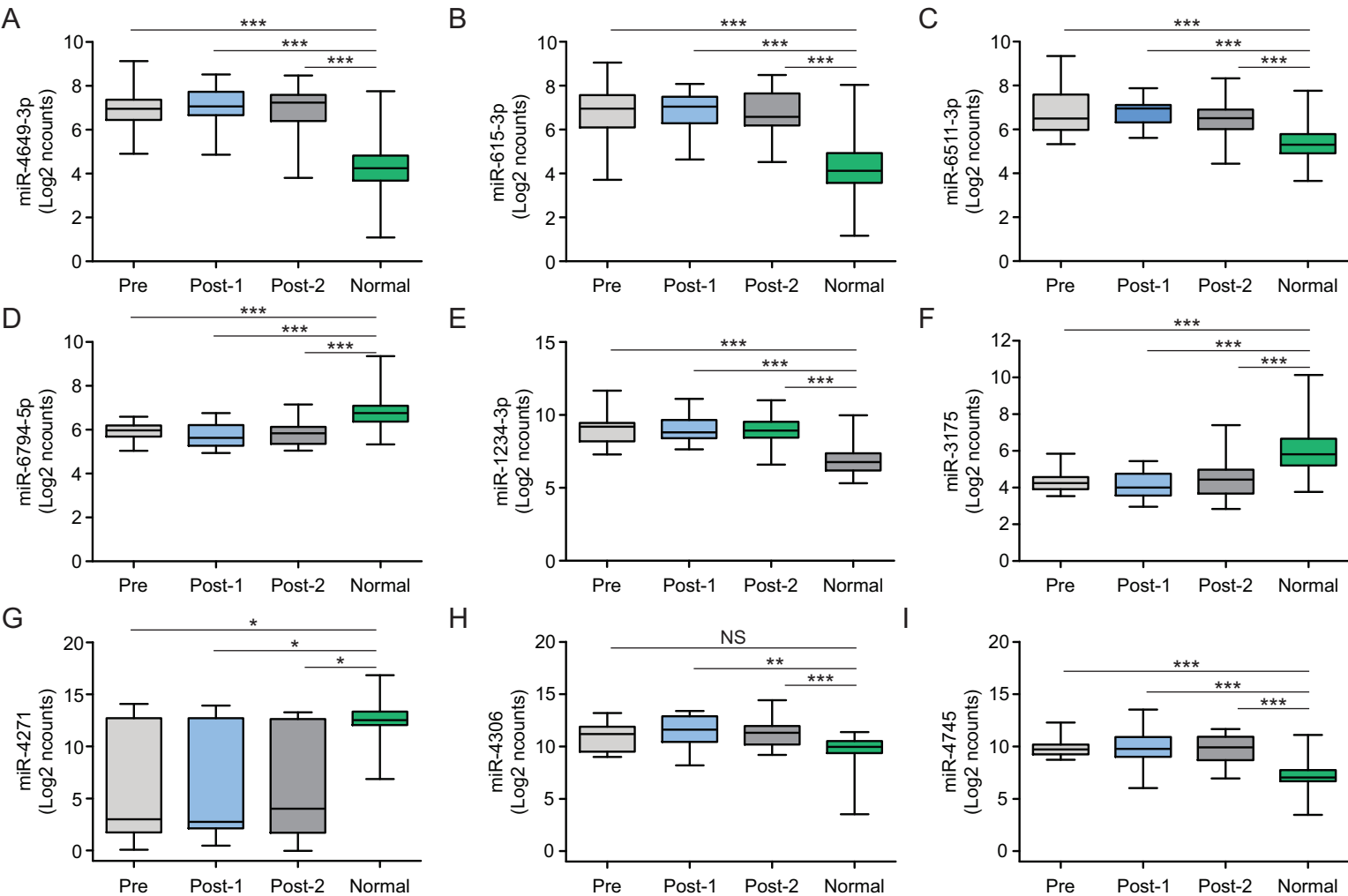

Supplement: Supplementary file 1 [file cancers-12-03361-s001.zip › cancers-1007326-XML suppl/Figure S4.pdf]

Figure S5

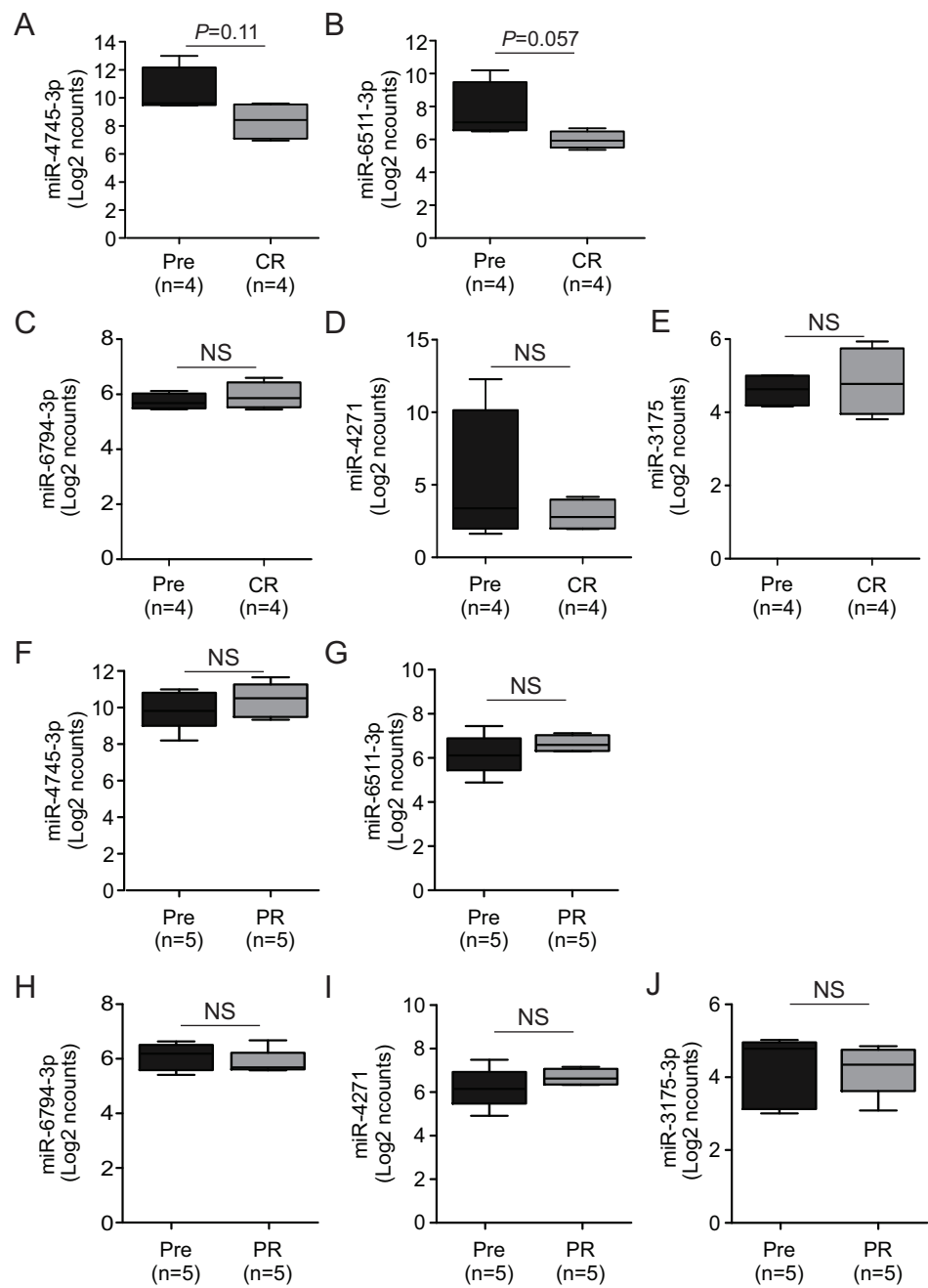

Supplement: Supplementary file 1 [file cancers-12-03361-s001.zip › cancers-1007326-XML suppl/Figure S5.pdf]

Figure S6

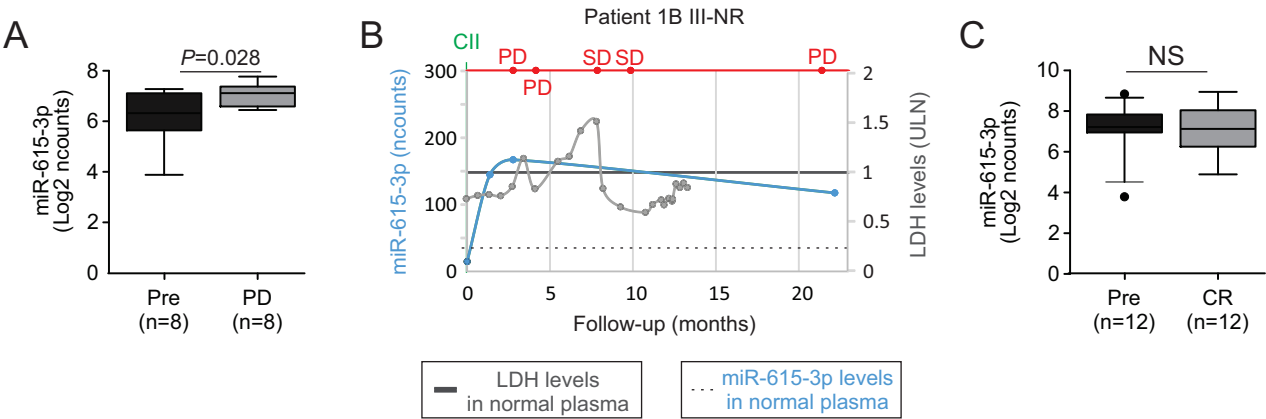

Supplement: Supplementary file 1 [file cancers-12-03361-s001.zip › cancers-1007326-XML suppl/Figure S6.pdf]

Figure S7

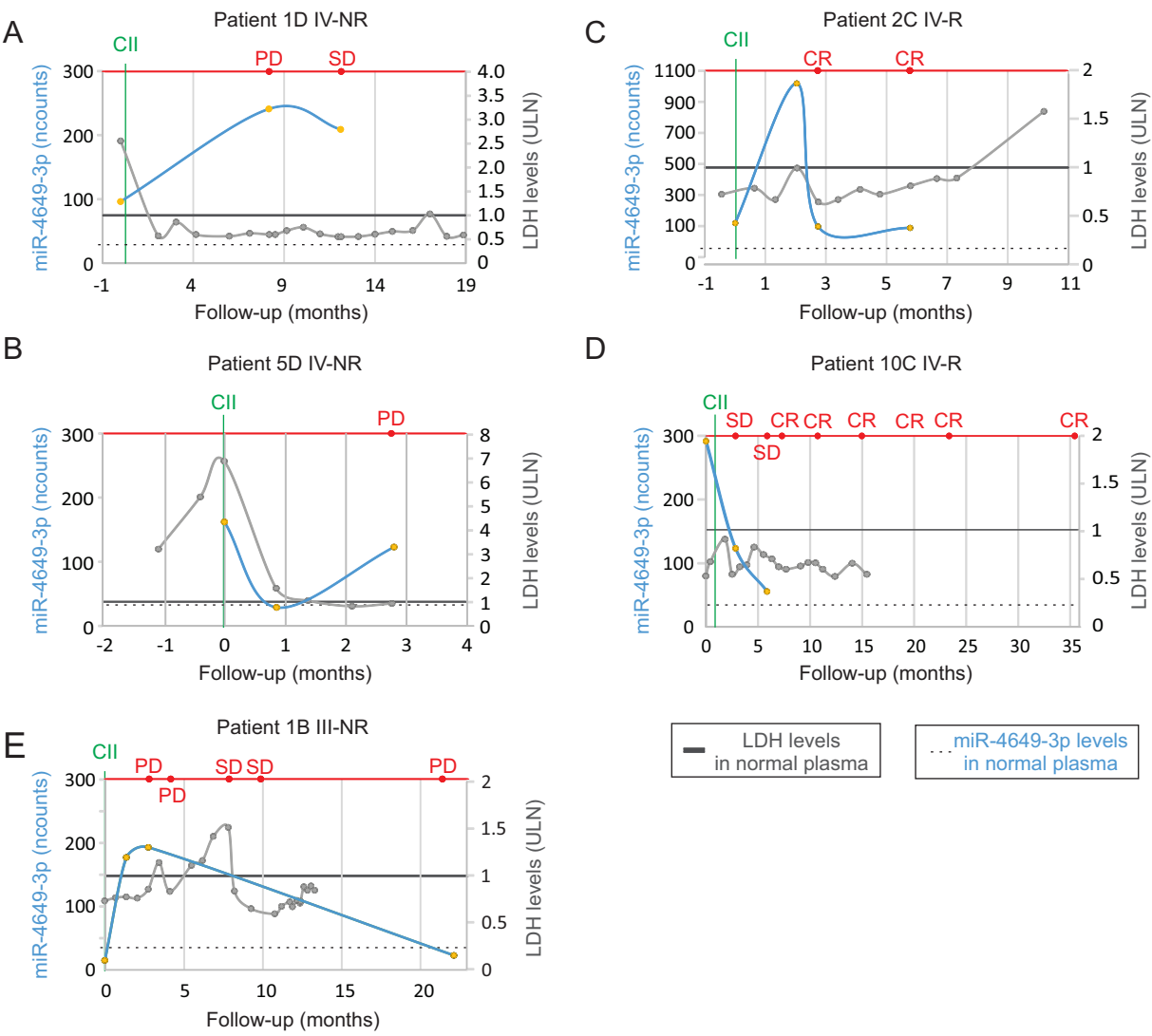

Supplement: Supplementary file 1 [file cancers-12-03361-s001.zip › cancers-1007326-XML suppl/Figure S7.pdf]
